# Cellular and Enzymatic Determinants Impacting the Exolytic Action of an Anti-Staphylococcal Enzybiotic

**DOI:** 10.3390/ijms25010523

**Published:** 2023-12-30

**Authors:** Ana Gouveia, Daniela Pinto, Jorge M. B. Vítor, Carlos São-José

**Affiliations:** 1 Phage Biology Research and Infection Control (PhaBRIC), Research Institute for Medicines (iMed.ULisboa), Faculdade de Farmácia, Universidade de Lisboa, Av. Prof. Gama Pinto, 1649-003 Lisboa, Portugal; ana.isabel.gouveia@ff.ulisboa.pt (A.G.); dspinto@ciencias.ulisboa.pt (D.P.); 2 Pathogen Genome Bioinformatics and Computational Biology, Research Institute for Medicines (iMed.ULisboa), Faculdade de Farmácia, Universidade de Lisboa, Av. Prof. Gama Pinto, 1649-003 Lisboa, Portugal; jvitor@ff.ulisboa.pt

**Keywords:** endolysin, lysin, enzybiotic, peptidoglycan hydrolase, cell wall, antibiotic resistance, membrane potential, proton motive force, wall teichoic acids, *Staphylococcus aureus*

## Abstract

Bacteriophage endolysins are bacteriolytic enzymes that have been explored as potential weapons to fight antibiotic-resistant bacteria. Despite several studies support the application of endolysins as enzybiotics, detailed knowledge on cellular and enzymatic factors affecting their lytic activity is still missing. The bacterial membrane proton motive force (PMF) and certain cell wall glycopolymers of Gram-positive bacteria have been implicated in some tolerance to endolysins. Here, we studied how the anti-staphylococcal endolysin Lys11, a modular enzyme with two catalytic domains (peptidase and amidase) and a cell binding domain (CBD_11_), responded to changes in the chemical and/or electric gradients of the PMF (ΔpH and Δψ, respectively). We show that simultaneous dissipation of both gradients enhances endolysin binding to cells and lytic activity. The collapse of ΔpH is preponderant in the stimulation of Lys11 lytic action, while the dissipation of Δψ is mainly associated with higher endolysin binding. Interestingly, this binding depends on the amidase domain. The peptidase domain is responsible for most of the Lys11 bacteriolytic activity. Wall teichoic acids (WTAs) are confirmed as major determinants of endolysin tolerance, in part by severely hindering CBD_11_ binding activity. In conclusion, the PMF and WTA interfere differently with the endolysin functional domains, affecting both the binding and catalytic efficiencies.

## 1. Introduction

Antimicrobial resistance (AMR) is a leading cause of death worldwide [1], and its continued rise constitutes a major threat to human health and the global economy [2,3,4]. As innovation regarding conventional (small-molecule) antibiotics has been mostly relying on the modification of established classes [5], truly alternative antimicrobials are needed, preferentially more pathogen-specific and with new modes of action that minimize resistance development. Among such alternatives are bacteriophage (phage) lytic enzymes, namely endolysins and engineered derivatives [6]. These enzymes destroy the bacterial cell wall (CW) and are viewed as a promising novel class of antimicrobials (enzybiotics) [7,8]. Some have already reached clinical trials [9].

At the end of bacterial infection, phages employ endolysins to degrade a major structural component of the host CW, the peptidoglycan, resulting in osmotic cell lysis for virion progeny release. Another phage-encoded protein instrumental to lysis is the holin, a protein that causes a fatal dissipation of the proton motive force (PMF) by forming “holes” in the host cytoplasmic membrane. The holin channels can have a dual role: to provide a pathway for the passage of the endolysin from the cytoplasm to the CW and/or to activate the lytic action, through PMF dissipation, of endolysins (pre)positioned in the CW compartment [10,11]. The PMF across the cytoplasmic membrane, which is crucial for cell survival, consists of two components: the electrical potential (Δψ) and the proton gradient (ΔpH) [12]. To maintain PMF homeostasis, bacteria can tune the relative contribution of Δψ and ΔpH in response to changes in growth conditions [13,14].

In addition to peptidoglycan, other glycopolymers make part of the CW of Gram-positive bacteria, among which teichoic acids (TAs) are perhaps the best-studied ones [15]. These can be covalently linked to the peptidoglycan mesh or be attached to the membrane via a lipidic anchor, being designated wall teichoic acids (WTAs) and lipoteichoic acids (LTAs), respectively. TAs play several functions in bacterial physiology, including protection against antibacterial agents (e.g., bacteriocins, antimicrobial peptides and certain antibiotics) and the control of endogenous enzymes involved in peptidoglycan synthesis, cleavage and cell division. These functions can be modulated by the decorations of the glycopolymers such as glycosylation and the incorporation of D-alanine esters, the latter of which are thought to mask negatively charged sites in TAs [16,17].

The capacity of endolysins to cleave the CW peptidoglycan when exogenously added to bacteria is at the basis of their intense exploration as enzybiotics. It is usually considered that Gram-positive bacteria are more susceptible to endolysin attack than Gram-negative and mycobacteria because these have an outer membrane that hinders enzyme access to the peptidoglycan [10,18]. However, it has been observed for different Gram-positive bacteria growing in nutrient-rich environments that they can display different levels of tolerance to endolysins. The mechanisms responsible for this tolerance are still poorly understood, but they were shown to rely on an operational PMF and on the presence of certain CW glycopolymers. Studies have demonstrated that agents that dissipate both PMF components, such as membrane ionophores, holins and cationic peptides, can render Gram-positive bacteria much more susceptible to endolysin attack [19,20,21]. In fact, the membrane- and peptidoglycan-acting agents can act synergistically to promote cell killing [22,23,24]. On the other hand, WTAs were shown to greatly restrict the action of lytic enzymes, including endolysins, at least in part by hindering their binding/access to the CW peptidoglycan [24,25,26,27].

This study aimed at increasing our knowledge on the determinants of endolysin tolerance, using as a model the endolysin Lys11 that targets an important Gram-positive pathogen in the context of AMR, *Staphylococcus aureus*. Lys11 has one of the most common domain architectures found in staphylococcal endolysins, displaying from N- to C-terminus a CHAP peptidase domain (CHAP_11_), an Amidase domain (Ami_11_) and an SH3-like cell binding domain (CBD_11_) [28]. We used selective membrane drugs to decompose the relative contribution of each PMF component to tolerance. *S. aureus* mutants were also employed to study if, in addition to the WTA, other CW components and/or modifications played a role in endolysin susceptibility. The impact of these cellular cues was evaluated on the whole enzyme and its individual functional domains, monitoring lytic and cell binding activities. We show that the two PMF gradients produce distinct effects in endolysin functional domains, impacting their binding and peptidoglycan cleavage activities. We also provide a clearer view of WTAs as restrictors of endolysin binding.

## 2. Results

### 2.1. The ΔpH Component of the PMF Has Major Contribution to Endolysin Tolerance

As mentioned in Section 1, the membrane PMF integrates the Δψ and ΔpH components, i.e., the electrical and proton gradients, respectively. Gramicidin, which collapses both gradients by forming channels in the membrane [12,29], is amongst the PMF-dissipating agents previously shown to significantly increase *S. aureus* susceptibility to the lytic action of endolysin Lys11 [21]. To understand the relative contribution of each PMF component to endolysin tolerance, *S. aureus* cells in rich culture media were treated with gramicidin, nigericin or valinomycin, before being challenged with Lys11. Nigericin promotes the electroneutral exchange of K^+^ for H^+^, whereas valinomycin promotes K^+^ influx under an external excess of this cation. Hence, the two latter agents were employed as selective ionophores dissipating ΔpH and Δψ, respectively [12,29,30]. We confirmed the expected effect of the three ionophores on the membrane potential and cell viability, using the membrane depolarization sensitive dye DiSC_3_(5) [24] and by determining colony-forming units per milliliter (CFU/mL), respectively (Figure S1).

**Figure 1:**
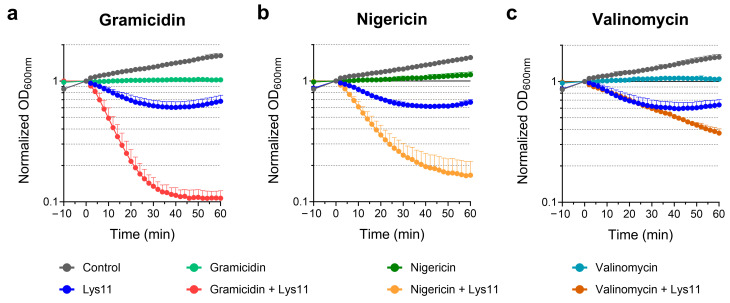
Bacteriolytic activity of endolysin Lys11 is enhanced upon PMF dissipation. Log phase cells of *S. aureus* strain RN4220 were collected in TSB medium supplemented with 0.5 mM CaCl2 (TSBca) and treated for 10 min with 30 µg/mL gramicidin (**a**), 10 µM nigericin (**b**) or 20 µM valinomycin (**c**). Cells treated with valinomycin were first supplemented with 200 mM KCl. After ionophore treatment, 100 nM Lys11 was added to cells, and lysis was monitored by following the optical density at 600 nm (OD_600nm_). Ionophore solvents and endolysin buffer were added to “Control” curves. Endolysin buffer was added to cells treated with the ionophore only. Time points −10 and 0 min indicate the time of ionophore and endolysin addition, respectively. OD_600nm_ values were normalized at t = 0 min. Each curve represents means ± standard deviation from at least 4 independent experiments. For clarity, only the mean + standard deviation is represented.

After 10 min treatment of *S. aureus* with each ionophore, Lys11 was added to cells, and lysis was monitored by following the culture optical density at 600 nm (OD_600nm_). In agreement with previous observations, gramicidin treatment clearly turned cells more susceptible to Lys11 lysis. For the tested Lys11 concentration (100 nM), culture OD_600nm_ decreased by almost 90% in gramicidin-treated cells, within 40 min, whereas for the same contact time, they were only reduced by ~40% in the presence of the endolysin alone (Figure 1a). Nigericin also provoked an obvious stimulation of Lys11-mediated lysis, although it was somewhat slower and less extensive compared to gramicidin (Figure 1b). Valinomycin was less effective in potentiating endolysin lytic action, with cultures lysing at a much slower pace (~60% lysis after 60 min, Figure 1c). In the assay conditions, the ionophores inhibited cell growth without causing visible cell lysis (Figure 1).

Taken together, the results indicated that although maximum endolysin susceptibility is achieved upon dissipating both PMF components, the pH gradient across the membrane seems to have a preponderant role in antagonizing Lys11 lytic action.

### 2.2. Dissipation of Both PMF Components Stimulates Endolysin Binding to Cells

In Section 2.1, we showed that the dissipation of both PMF components (gramicidin) or of the ΔpH only (nigericin) turned *S. aureus* cells significantly more prone to lysis by Lys11. Since previous studies (see Section 1) have shown that tolerance to lytic enzymes could be associated with a deficient binding of the proteins to the cell surface, we questioned whether the higher cell susceptibility to Lys11 in the presence of the ionophores correlated with an increased endolysin binding to cells. To address this question, we fused different Lys11 domains to the enhanced green fluorescent protein (eGFP) and measured their binding to *S. aureus* cells treated with the ionophores. The constructed fusions were eGFP-Ami_11_-CBD_11_, eGFP-Ami_11_ and eGFP-CBD_11_ (Supplemental File S1 and Figures S2 and S3). Note that although an amidase activity has been attributed to Ami_11_ in studies with purified CW [28], in Lys11 and some related enzymes, the amidase domain was shown to contribute poorly to exolytic activity against intact cells. However, it was found to significantly enhance endolysin binding to the *S. aureus* surface when associated with the cell binding domain [24,31]. Therefore, the binding of eGFP-Ami_11_-CBD_11_ was assumed here to be a proxy of Lys11 binding, whereas the two other fusions were intended to provide information regarding the relative contribution of Ami_11_ and CBD_11_ to the binding. Since Lys11 derivatives carrying CHAP_11_ as the sole catalytic domain can cause significant cell lysis in certain conditions (see Section 2.6), measuring by the same approach the possible contribution of this domain to binding was not straightforward, and therefore it was not investigated in this study.

**Figure 2:**
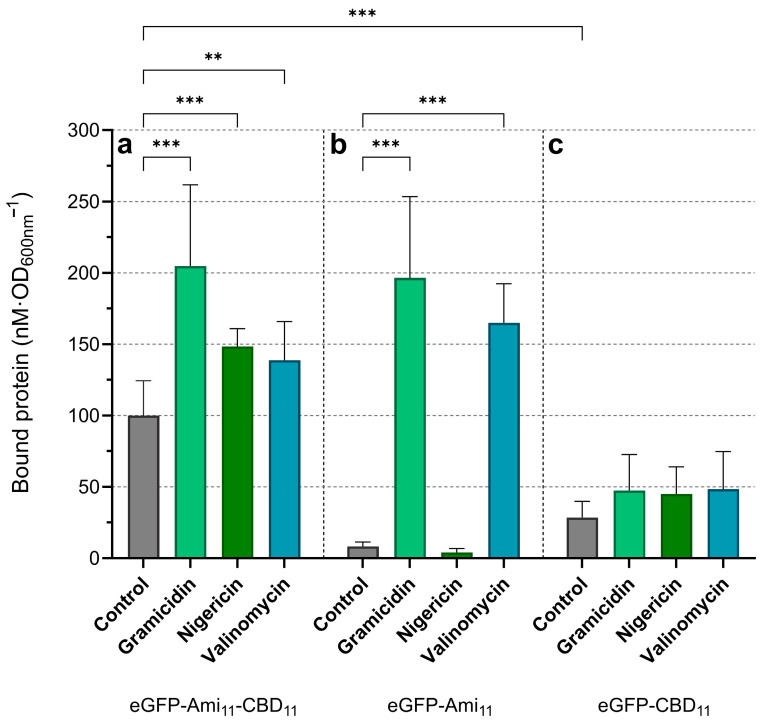
Lys11 binding to *S. aureus* cells is enhanced by collapsing the PMF. Cells of strain RN4220 harvested in TSBca were treated with gramicidin, nigericin, valinomycin or ionophore solvent (“Control”) as in Figure 1. Following treatment, 100 nM of eGFP-Ami_11_-CBD_11_ (**a**), eGFP-Ami_11_ (**b**) or eGFP-CBD_11_ (**c**) was added to samples and further incubated for 10 min. After removal of free protein, the amount of eGFP fusion bound to cells was quantified by fluorimetry (see Section 4.5). The data represent means ± standard deviation from at least 6 independent experiments. Asterisks denote significant differences according to two-way ANOVA test, followed by Tukey post hoc test (** *p* < 0.01; *** *p* < 0.001).

*S. aureus* cells were exposed or not to the ionophores as described above and then incubated with the eGFP fusions. After removing the unbound protein, the amount of eGFP fusion associated with cells was quantified by fluorimetry as described in Gouveia et al. [24] (see Section 4.5). The binding of eGFP-Ami_11_-CBD_11_ was enhanced after collapsing both PMF gradients with gramicidin, resulting in ~2-fold more bound protein when compared to untreated, control cells (Figure 2a). Selective dissipation of either ΔpH (nigericin) or Δψ (valinomycin) also promoted eGFP-Ami_11_-CBD_11_ binding to cells, although it was not as pronounced as gramicidin (~1.4-fold increase). The fact that nigericin was not as effective as gramicidin in stimulating endolysin binding could contribute to the lower Lys11 lytic performance in response to nigericin when compared to gramicidin (Figure 1a,b).

The fusion eGFP-Ami_11_ bound poorly both to control and nigericin-treated cells (Figure 2b). Remarkably, its binding was drastically increased upon gramicidin and valinomycin treatment (~23- and ~20-fold increase relative to control cells, respectively). Finally, eGFP-CBD_11_ showed moderate binding to control cells (Figure 2c), which was about 1/3 of that measured with eGFP-Ami_11_-CBD_11_ in the same condition (Figure 2a). Cells treated with any of the ionophores appeared to register some increase in eGFP-CBD_11_ binding (Figure 2c), although it was not considered statistically significant probably due to the relatively large standard deviations.

Overall, the results indicated that PMF dissipation favored endolysin binding to cells, which could at least partially explain the higher Lys11 lytic action in these conditions. This increased endolysin binding appears to derive in great part from a stimulation of Ami_11_ binding activity in response to PMF collapse, particularly the elimination of the Δψ component (Figure 2b). Yet, the isolated Ami_11_ bound poorly to energized cells (Figure 2b, Control), while it stimulated binding when associated with CBD_11_ (seen when we compare the binding of eGFP-Ami_11_-CBD_11_ and eGFP-CBD_11_ to control cells). This hints at either a cooperative binding of Ami_11_ and CBD_11_ when in the same polypeptide chain or an indirect role of Ami_11_ as an enhancer of the proper CBD_11_ conformation for binding.

### 2.3. PMF Dissipation Simultaneously Favors Endolysin Binding and Peptidoglycan Cleavage

**Figure 3:**
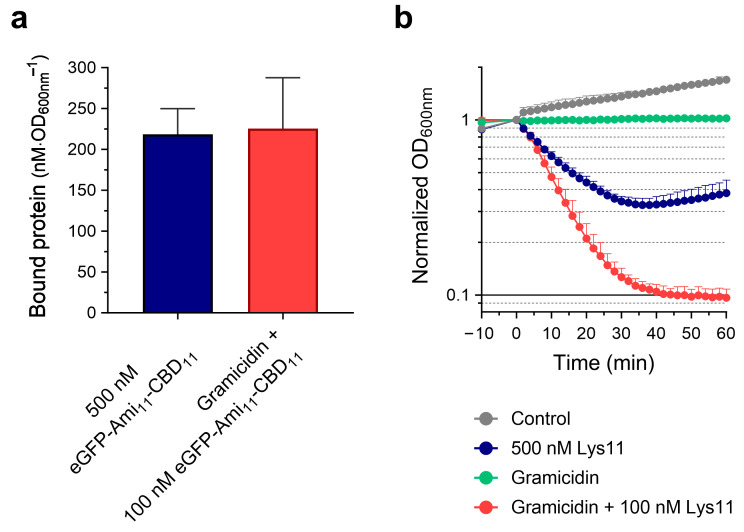
PMF dissipation enhances Lys11 lysis by simultaneously favoring endolysin binding to cells and peptidoglycan cleavage. (**a**) Cells of strain RN4220 were untreated or treated with gramicidin and then incubated with 500 and 100 nM of eGFP-Ami_11_-CBD_11_, respectively, which resulted in similar amounts of fluorescent protein bound to cells (no significant differences of bound protein according to Student’s *t*-test). The data represent means ± standard deviation from at least 9 independent experiments. (**b**) In the corresponding lysis assay, 500 and 100 nM of Lys11 were added to cells that had been untreated or treated with gramicidin, respectively. Despite the similar endolysin binding inferred in (**a**), the condition gramicidin + 100 nM Lys11 causes more extensive and faster cell lysis. The data represent means ± standard deviation from at least 5 independent experiments.

The ensemble of results from Section 2.1 and Section 2.2 suggested that the collapse of both PMF gradients potentiated Lys11 bacteriolytic action in two different ways: by favoring endolysin binding to cells and by stimulating peptidoglycan cleavage. The latter effect was mainly inferred after the dissipation of ΔpH (nigericin), which clearly improved Lys11 bacteriolytic action (Figure 1b) while causing only a moderate increase in eGFP-Ami_11_-CBD_11_ binding (Figure 2a). To further support this dual effect, we devised an experiment in which depolarized and energized cells would bind similar amounts of eGFP-Ami_11_-CBD_11,_ followed by an evaluation of the corresponding lysis profiles with Lys11. The incubation of untreated cells with 500 nM of eGFP-Ami_11_-CBD_11_ resulted in amounts of bound protein similar to those obtained after the incubation of gramicidin-treated cells with 100 nM of the fluorescent protein (Figure 3a). Yet, in the corresponding lysis assays with Lys11, the condition gramicidin plus 100 nM endolysin produced much faster and more extensive cell lysis (Figure 3b). This was another indication that the enhancement of endolysin binding in response to PMF collapse is not sufficient to explain the highest Lys11 lytic action and that a stimulation of peptidoglycan cleavage should also be occurring. This increased cleavage may result from an effect produced either in the substrate (peptidoglycan) and/or in the enzyme’s catalytic activity upon PMF collapse.

### 2.4. WTA Is the Key CW Glycopolymer Contributing to Endolysin Tolerance

As referred to in Section 1, CW glycopolymers and their modifications can impact the action of peptidoglycan-degrading enzymes. Under conditions supporting bacterial growth, WTAs were shown to hinder the binding of lytic enzymes to the CW surface [25,26,27], including Lys11 [24]. In this work, we wanted to test if LTA, the other major glycopolymer associated with the *S. aureus* CW, could also play a role in cell susceptibility to Lys11 lysis. In addition, we sought to investigate the possible influence of major TA modifications. For this, we used an *S. aureus* mutant disabled in LTA synthesis (Δ*ltaS*) that is still capable of almost normal growth due to the presence of a suppressor mutation in the GdpP phosphodiesterase [32]. For the TA modifications, we used a double mutant Δ*tarM*Δ*tarS*, which is simultaneously impaired in α-O-GlcNAcylation and β-O-GlcNAcylation of WTA [33], and a Δ*dltA* mutant that cannot perform D-alanylation of TAs [34]. All mutants were derivatives of the *S. aureus* strain RN4220 used throughout this work, except the Δ*dltA* mutant that was derived from strain SA113.

**Figure 4:**
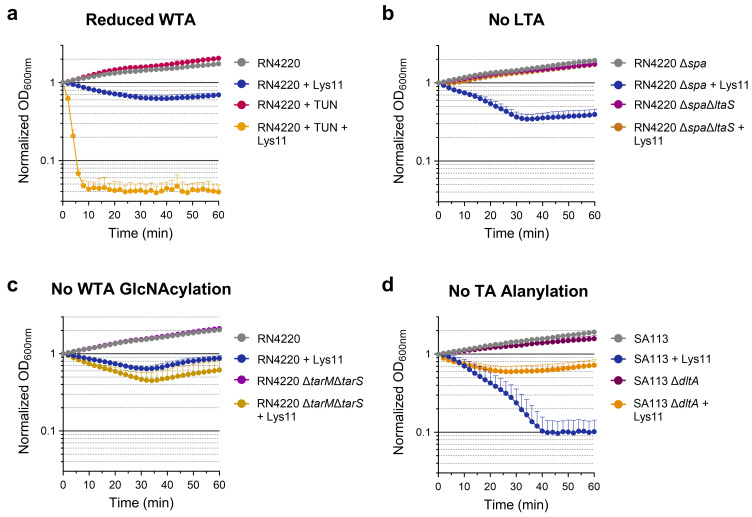
Contribution of CW glycopolymers and their modifications to Lys11 tolerance. Log phage cells of the indicated *S. aureus* strains and derived mutants were collected in TSBca and then challenged with 100 nM Lys11. Bacteriolysis was followed by taking OD_600nm_ measurements. (**a**) Cells grown in absence or presence of 50 ng/mL tunicamycin (TUN), which reduces WTA synthesis without affecting growth. (**b**) Mutant Δ*spa*Δ*ltaS* is impaired in LTA synthesis, whereas the control strain Δ*spa* has normal LTA production. (**c**) The mutant Δ*tarM*Δ*tasS* lacks the α- and β-O-GlcNAcylation modification of WTA. (**d**) The Δ*dltA* mutation causes no alanylation of TAs. Each curve represents means ± standard deviation from 5 independent experiments. For clarity, only the mean + standard deviation is represented.

By growing cells in the presence of an inhibitor of WTA synthesis (tunicamycin), we confirmed that in our experimental conditions, the *S. aureus* WTA works as an important determinant of Lys11 tolerance [24]. A less than 10 min contact of Lys11 with tunicamycin-grown cells was sufficient to cause more than 90% cell lysis (Figure 4a). The Δ*ltaS* mutant was constructed in an RN4220Δ*spa* background, which exhibits normal LTA production but lacks protein A (SpA) in the CW surface [32]. The growth of the mutant RN4220Δ*spa*Δ*ltaS* was unaffected in the presence of Lys11 (Figure 4b), therefore displaying even higher endolysin tolerance than strain RN4220 (Figure 4a). Curiously, the intermediate strain RN4220Δ*spa* seemed more susceptible to Lys11 attack than RN4220, with culture OD_600nm_ decreasing almost 70% within 40 min (Figure 4b). This hinted at some antagonizing effect of SpA toward Lys11 lytic action, something we did not explore in this work. Nevertheless, the increased tolerance of the mutant RN4220Δ*spa*Δ*ltaS* to Lys11 was an indication that the *S. aureus* LTA should not work as restrictor of the endolysin bacteriolytic activity.

Regarding the mutants affected in TA modifications, we found that the lack of α- and β-O-GlcNAcylation of WTA resulted, at most, in a slight increase in *S. aureus* susceptibility to Lys11 lytic action (Figure 4c), indicating that these substitutions have no major role in the endolysin antagonistic action of WTA. The Δ*dltA* mutant (no TA alanylation) had much lower endolysin susceptibility than the parental strain SA113 (Figure 4d). Interestingly, SA113 was previously described as a low-WTA *S. aureus* strain [35], which should explain its considerably higher vulnerability to Lys11 lysis compared to strain RN4220. Despite this, the results obtained with the Δ*dltA* mutant indicated that normal TA D-alanylation favors Lys11 lysis, instead of conferring protection, at least in strain SA113.

Overall, the assays with *S. aureus* cells affected in TA composition confirmed WTA as the major CW glycopolymer contributing to endolysin tolerance.

### 2.5. WTA Drastically Hinders Binding Meditated by CBD11

**Figure 5:**
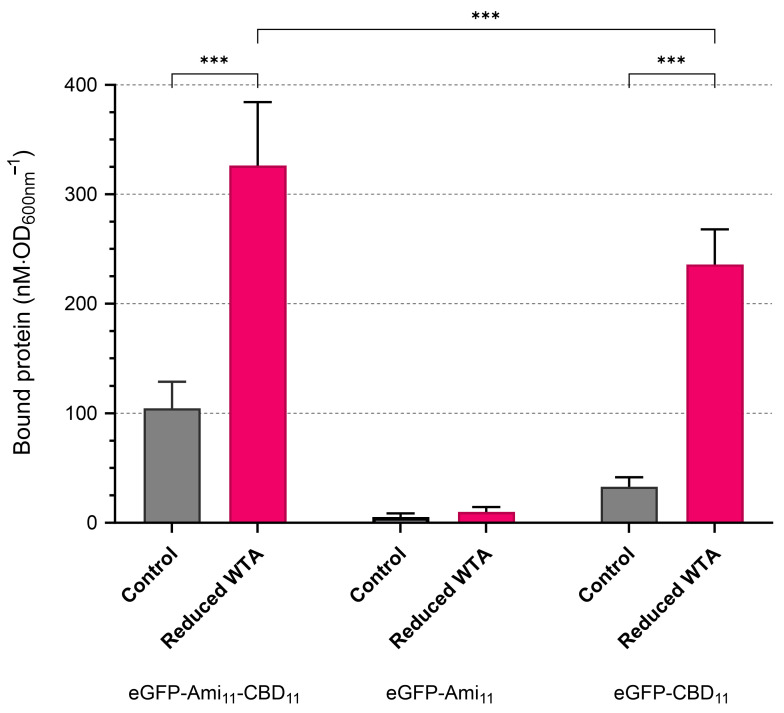
WTA primarily affects CBD_11_ binding. Log phase cells of strain RN4220 grown in absence (“Control”) or presence of 50 ng/mL tunicamycin (“Reduced WTA”) were collected in TSBca and, after 10 min incubation with the indicated eGFP-endolysin fusions, the amount of fluorescent protein associated with cells was determined as in Figure 2. The data represent means ± standard deviation from at least 7 independent experiments. Asterisks denote a significant difference, according to two-way ANOVA test, followed by Tukey post hoc test (*** *p* < 0.001).

Next, we studied how WTA interfered with the binding efficiency of the three eGFP fusions used in Section 2.2, by quantifying their association with cells grown in the presence or absence of tunicamycin. In agreement with previous observations [24], the fusion eGFP-Ami_11_-CBD_11_ bound approximately three times more efficiently to cells with diminished WTA content (Figure 5). The binding of eGFP-Ami_11_ was again minimal and not significantly augmented by reducing the WTA level in the CW. Notably, eGFP-CBD_11_ binding to cells with low WTA content was ~7-fold higher than the binding to cells with normal WTA production (Figure 5). Taken together, the results indicated that CBD_11_ has a dominant role in endolysin binding to cells with low WTA. In agreement with this, the binding of eGFP-Ami_11_-CBD_11_ to cells with inhibited WTA synthesis was only ~1.4-fold higher than that of eGFP-CBD_11_.

In summary, the results strongly suggested that WTA restricts Lys11 association to the CW mainly by interfering with the CBD_11_ binding activity. Also, the binding auxiliary role of the amidase domain seems to be more relevant for Lys11 association with cells with normal WTA content (Figure 2a,b).

### 2.6. Lys11 Catalytic Domains Respond Differently to Cell Tolerance Determinants

Finally, we wanted to understand how the Lys11 catalytic domains (CDs), CHAP_11_ and Ami_11_, responded in terms of lytic activity to the changes in the cell membrane energetic state (using the ionophores) and to the WTA reduction in the CW (achieved with tunicamycin). For that, we produced Lys11 derivatives having each CD fused to the cell binding domain, i.e., CHAP_11_-CBD_11_ and Ami_11_-CBD_11_ (Supplemental File S1 and Figures S2 and S3).

**Figure 6:**
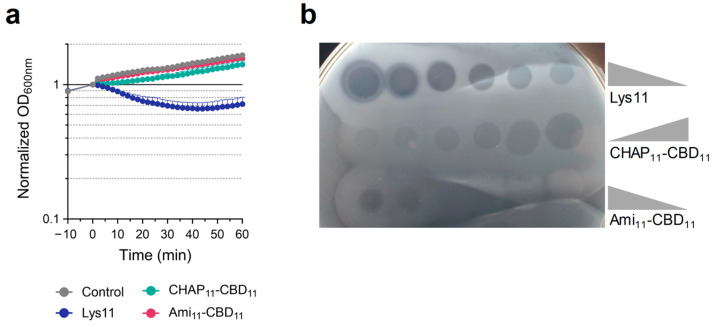
Lytic action of single-CD mutants of Lys11. (**a**) Cells of strain RN4220 in TSBca were incubated for 10 min with ionophore solvent, and then 100 nM of the indicated lysin variants was added. Cell lysis was monitored by following culture OD_600nm_. Each curve represents means ± standard deviation from 6 independent experiments. For clarity, only mean + standard deviation is represented. (**b**) The lytic activity of Lys11, CHAP_11_-CBD_11_ and Ami_11_-CBD_11_ was evaluated by spotting 10 µL of 2-fold serial dilutions of the proteins on a dense lawn of *S. aureus* RN4220 (see Section 4.4). Lysin concentrations ranged from 5 to 0.16 µM. Note that the whitish opacity around lysis halos is due to the high glycerol concentration (30%) present in the lysin storage buffer, which is also serially diluted like the proteins.

The lytic action of Lys11 and its CD deletion mutants was again tested at 100 nM. As seen in Section 2.1, at this concentration, Lys11 stopped *S. aureus* growth and caused some cell lysis. In contrast, the single-CD mutants could not affect culture growth, with the corresponding curves being essentially indistinguishable from that of the control cells (Figure 6a). To verify that CHAP_11_-CBD_11_ and Ami_11_-CBD_11_ retained peptidoglycan degrading activity, they were serial diluted and spot-tested on a dense lawn of viable *S. aureus* cells incorporated in a buffered, soft agar matrix (see Section 4.4). From our experience, this is one of the most sensitive assays to reveal lysin lytic activity. In these conditions, CHAP_11_-CBD_11_ appeared to be just slightly less bacteriolytic than Lys11, as judged by the somewhat clearer lysis halos of the latter (Figure 6b). In contrast, an obvious Ami_11_-CBD_11_ lytic effect could only be observed at the two highest spotted concentrations (5 and 2.5 µM). As already noted, a reduced peptidoglycan cleaving activity of the amidase domain of Lys11 and some related endolysins was previously described [28,31,36]. Thus, the results indicated that the single-CD mutants could not elicit obvious cell lysis of liquid cultures, despite displaying significant (CHAP_11_-CBD_11_) or residual (Ami_11_-CBD_11_) peptidoglycan-degrading activity in the spot assay.

**Figure 7:**
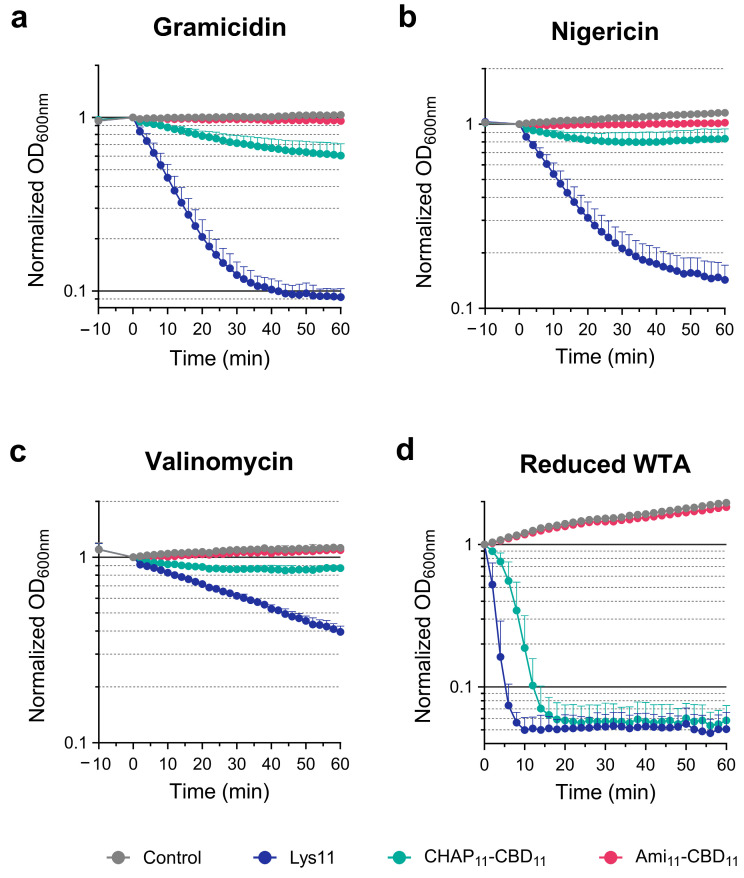
The catalytic domains of Lys11 respond differently to cell tolerance signals. Cells of strain RN4220 in TSBca were treated with gramicidin (**a**), nigericin (**b**) or valinomycin (**c**). Following ionophore treatment, 100 nM of the indicated proteins were added, and cell lysis was monitored. (**d**) Cells of strain RN4220 grown in presence of tunicamycin were collected in TSBca, they were challenged with 100 nM of the indicated proteins, and then cell lysis was similarly monitored. In each panel, the “Control” curves correspond to cells with ionophore or tunicamycin only (no protein added). Each curve represents means ± standard deviation from at least 4 independent experiments. For clarity, only the mean + standard deviation is represented.

When CHAP_11_-CBD_11_ was tested against cells treated with gramicidin, which affects both PMF components, slow but steady cell lysis could be observed, with culture OD_600nm_ being reduced by ~40% after 60 min (Figure 7a). In the same conditions, no cell lysis could be measured with Ami_11_-CBD_11_ (Figure 7a), even when its concentration was increased to 4 µM (Figure S4). Therefore, the poor lytic performance of the single-CD mutants in liquid cultures remained unaffected (Ami_11_-CBD_11_) or showed some stimulation (CHAP_11_-CBD_11_) after PMF collapse, contrasting with Lys11 that lysed ~90% of the cells within 40 min, as seen before. As expected, the ΔpH and Δψ selective ionophores (nigericin and valinomycin, respectively) failed to significantly potentiate the lysis by the single-CD mutants, with CHAP_11_-CBD_11_ only causing a slight drop in the OD_600nm_ curve when compared to those of Ami_11_-CBD_11_ and cells with the ionophore only (Figure 7b,c).

**Figure 8:**
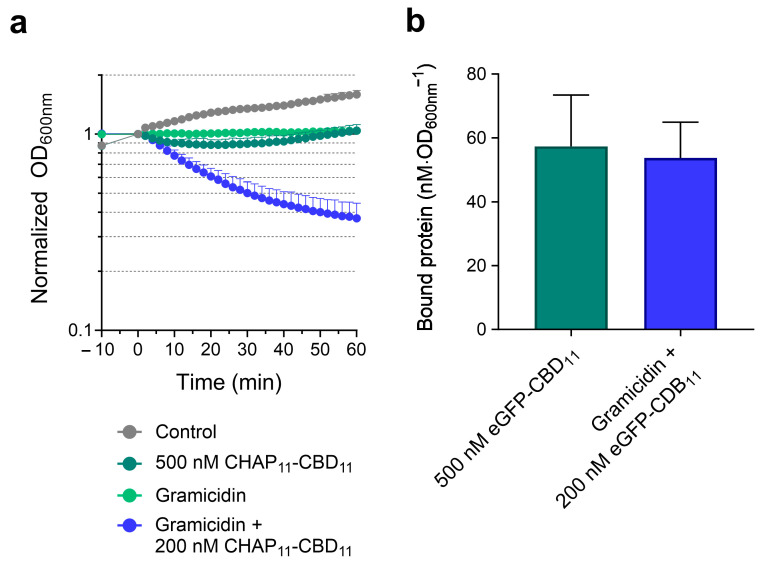
PMF dissipation with gramicidin facilitates peptidoglycan cleavage by CHAP_11_-CBD_11_. (**a**) Cells of strain RN4220 were untreated or treated with gramicidin and then incubated with 500 and 200 nM of eGFP-Ami_11_-CBD_11_, respectively. Only the condition gramicidin + 200 nM Lys11 caused obvious cell lysis. The data represent means ± standard deviation from at least 5 independent experiments. (**b**) In the corresponding binding assay with eGFP-CBD_11_, the two conditions resulted in similar amounts of fluorescent protein bound to cells (no significant difference according to Student’s *t*-test). The data represent means ± standard deviation from 7 independent experiments.

Hence, the results indicated that the two Lys11 CDs are required for efficient lysis and effective stimulation of the endolysin lytic action in response to PMF dissipation. Nevertheless, the spot (Figure 6b) and lysis assays (Figure 7a–c) denoted a preponderant role of CHAP_11_ in Lys11 lytic action. The fact that in the tested conditions CHAP_11_-CBD_11_ appeared to inefficiently respond to PMF dissipation could in part result from its poor binding to cells, due to the lack of the Ami_11_ stimulatory role in binding (eGFP-Ami_11_-CBD_11_ versus eGFP-CBD_11_, Figure 2). In fact, when the concentration of CHAP_11_-CBD_11_ was doubled (200 nM), the gramicidin enhancing effect on its lytic action became more evident, while 5-fold more protein (500 nM) still produced almost no lysis in the absence of the ionophore (Figure 8a). In addition, in the corresponding binding assays with eGFP-CBD_11_, these two conditions resulted in similar amounts of protein bound to cells (Figure 8b). Thus, as observed above for Lys11 (Figure 3), it can be inferred that for the same amount of bound CHAP_11_-CBD_11_ peptidoglycan cleavage is favored when cells are depolarized. Moreover, the equivalent binding in the two conditions hinted again at some improvement in the CBD_11_ binding efficiency to depolarized cells (see Figure 2c).

Finally, we tested the single-CD mutants on cells grown in the presence of tunicamycin (low WTA content). Although lysis mediated by Ami_11_-CBD_11_ remained undetectable in this condition, CHAP_11_-CBD_11_ lytic action was drastically improved, being able to reduce culture OD_600nm_ by ~90% in about 15 min (Figure 7d). This result confirmed that the poor lytic performance of CHAP_11_-CBD_11_ against depolarized cells, but with normal WTA content (Figure 7a), did not derive from a major intrinsic defect of the CHAP_11_ catalytic activity in the single-CD construct. Nevertheless, lysis by Lys11 was still the fastest (90% OD_600nm_ reduction in ~5 min) (Figure 7d), hinting again at a catalytic contribution of the Ami_11_ domain that is not apparent with the Ami_11_-CBD_11_. Note that as seen above, the Ami_11_ domain seemed to have only a moderate contribution to the binding to cells with reduced WTA content (Figure 5).

## 3. Discussion

**Figure 9:**
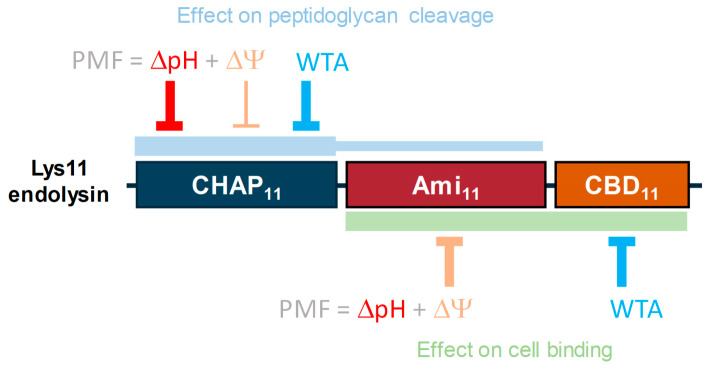
Schematic summary of the major findings of this work. The CHAP_11_ domain is responsible for most of the endolysin’s peptidoglycan cleavage activity, which is mainly restrained by the tolerance determinants ΔpH and WTA. An antagonist effect of WTA toward Lys11 cleavage activity was also inferred in a previous work [24]. The domains Ami_11_ and CBD_11_ cooperate in endolysin binding to the CW, being primarily counteracted by the tolerance determinants Δψ and WTA, respectively.

An increasing body of literature supports that Gram-positive bacteria under growth-supporting conditions can restrict to a certain extent the exolytic action of endolysins, something that might have implications in the application of native endolysins and engineered derivatives as enzybiotics [37]. This work aimed at contributing to our understanding of the endolysin tolerance phenomenon by studying in more detail previously known determinants, the PMF and WTAs, and by investigating new potential factors. As a model system, we used the bacterium *S. aureus* and the modular, dual-CD endolysin Lys11. The major findings are schematically summarized in Figure 9.

We started by dissecting the relative contribution of the two gradients that compose the PMF, ΔpH and Δψ, to endolysin tolerance. In our experimental conditions, the highest susceptibility to the Lys11 lytic action was observed in the presence of gramicidin, suggesting that both gradients of the PMF contribute to tolerance. Still, Lys11 lytic performance was clearly higher against cells treated with nigericin compared to valinomycin, indicating that the proton gradient (ΔpH) has a preponderant role in controlling susceptibility to Lys11. During bacterial growth, the pH gradient is generated by membrane pumps that extrude protons [14], creating a relatively low pH in the CW environment [38]. This acidification has been proposed as one of the mechanisms inhibiting the enzymatic activity of bacterial autolysins [39,40], and anionic polymers like WTA are thought to play an important role in this ΔpH inhibitory effect by retaining the extruded protons in the CW compartment [41,42]. Considering the structural and evolutionary relationship between bacterial autolysins and phage endolysins [43,44], it is not surprising that ΔpH also exerts control on the phage lytic enzymes, as we have shown here for Lys11.

The Lys11 deletion analysis indicated that most of the enzyme’s peptidoglycan cleavage activity relies on the CHAP_11_ domain, which is majorly restrained by the tolerance determinants ΔpH and WTA. Still, an auxiliary role of the Ami_11_ domain in peptidoglycan cleaving could be inferred from certain experiments. Such preponderant role of the CHAP CD in cutting the peptidoglycan, while the amidase CD seems to have a preeminent role in endolysin binding, is a consistent feature of this type of staphylococcal endolysin [31,45]. The full-length endolysin performed always better than the single-CD mutants, either under conditions supporting tolerance (cells growing in rich media) or favoring lysis (collapsed PMF or WTA deficiency), indicating that the native enzyme with its two CDs and a CBD is optimized for lytic activity. Our results agree with a previous study reporting that Lys11 constructs bearing either the endopeptidase or amidase domain directly fused to the CBD failed to induce visible lysis against heat-killed *S. aureus* [46]. However, in certain experimental conditions, structurally related staphylococcal endolysins were shown to display normal, or even superior, lytic activity, after the deletion of the amidase CD [45,47]. Therefore, for related endolysins, the outcome of combining or deleting functional domains can vary significantly, probably depending on the intrinsic features of the individual domains.

In agreement with previous suggestions [24], the data here presented showed that the PMF can influence endolysin affinity to the cell surface, which will naturally affect its lytic performance. The binding assays with the different eGFP-endolysin fusions showed that when Ami_11_ and CBD_11_ are in the same polypeptide chain, they contribute to maximizing binding both to normal and depolarized cells. The amount of eGFP-Ami_11_-CBD_11_ (the proxy of Lys11) that bound to cells with collapsed PMF was essentially doubled relative to normal cells (gramicidin-treated versus control cells). Interestingly, eGFP-Ami_11_ bound poorly to energized cells, but its binding was drastically increased after the abolishment of the Δψ gradient. Thus, during phage infection, it is likely that Ami_11_ favors Lys11 binding to the CW in response to the PMF collapse accomplished by the holin function.

In neutrophilic bacteria like *S. aureus* and *Bacillus subtilis*, the Δψ component of the PMF corresponds to an asymmetric distribution of ions across the membrane, with a net accumulation of cations outside the cell and anions inside [14]. The ionic environment of the CW together with its charged constituents determines the electrostatic binding properties of the bacterial surface. The collapse of the PMF can contribute to a more negative charge of the bacterial cell surface, resulting in an increased capacity to bind positively charged probes or molecules [39,41]. Our recombinant Lys11 and the fusion eGPF-Ami_11_-CBD_11_ have similar charge distributions and predicted positive net charges (+4.8 and +3.4, respectively) at pH 7.0, which is close to the medium pH in the assays. Therefore, the increased endolysin binding could be at least partially explained by an enhancement of the electrostatic interactions with the more negatively charged cell surface after PMF dissipation. Notably, the Ami_11_ module and its upstream linker contribute with most of the endolysin basic amino acids, having individual net charges of +7.3 and +2.8, respectively (Figure S2). The CHAP_11_ and CBD_11_ modules have net charges of −6.3 and +0.4, respectively, whereas the eGPF moiety in the fluorescent fusions has a net charge of −7.7. Thus, the cationic character of Ami_11_ could be at the basis of its highest responsiveness to Δψ collapse with respect to cell binding.

A recent study with two endogenous peptidases of *S. pettenkoferi* that have the same functional domains, but very distinct isoelectric points, showed that the affinity of the enzymes was governed by the protein and bacterial surface charges, with the relative lytic activities essentially correlating with the binding efficiencies [48]. In fact, modifications that increase lysin net positive charge have been proposed as a strategy to improve their association with the negatively charged bacterial surface and, with this, enhance exolysis (reviewed in [45]). However, such an approach does not always produce the desired effect [49], and in some cases, the neutralization of specific basic residues in the enzymes may actually increase processivity and lysis kinetics by allowing their faster dissociation from the CW [50]. In other words, excessive binding affinity may also be detrimental to lysis kinetics by restricting enzyme movement on the CW [50].

In this study, we evaluated the possible contribution of LTA and TA modifications to Lys11 tolerance. LTAs were proposed to have an inhibitory action against certain staphylococcal autolysins [51,52], whereas in some *S. aureus* genetic backgrounds, the presence of LTA seems to favor autolytic activity [53]. Hence, we questioned if LTA played any relevant role in Lys11 susceptibility. The *S. aureus* mutant disabled in LTA production used in this work showed complete tolerance to Lys11, whereas the parental strain, with normal LTA synthesis, presented some cell lysis in the same conditions. Therefore, the *S. aureus* LTA does not seem to contribute to Lys11 tolerance; on the contrary, it appears to facilitate Lys11 lytic action. The *S. aureus* LTA occupies the inner layers of the CW and is likely to preferentially accumulate in the site of its synthesis, the division septum [54]. The WTA tends to be excluded from this place and accumulate in the older regions of the CW [55]. Considering the key role of the WTA in Lys11 tolerance and that the nascent peptidoglycan at the septum might be more exposed to endolysin attack, a possible explanation for the lower susceptibility of the LTA mutant to Lys11 could be a compensatory accumulation of WTA [56], namely in the cell cross-wall.

The studies with *S. aureus* mutants deficient in WTA GlcNAcylation and TA D-alanylation indicated that these substitutions do not contribute significantly to Lys11 tolerance. The lack of α- and β-O-GlcNAcylation was previously reported to have little or no impact on *S. aureus* susceptibility to lysostaphin (only half-reduction in its MIC) and autolysin activity [33]. Yet, the substitution of TAs with cationic D-alanine esters has been shown to affect the activity of bacterial autolysins, lysostaphin and cationic antibacterial peptides. D-alanylation balances the negative character of WTAs and LTAs, and this was proposed to directly impact the binding of the referred-to agents to the CW and/or to change the local concentrations of cations that modulate their activity (reviewed in [16,17,57]). On this basis, and considering the predicted positive net charge of Lys11, one could expect higher endolysin binding to cells lacking TA D-alanylation and eventually increased exolysis. However, the susceptibility of the D-alanylation mutant to Lys11 lysis was clearly reduced compared to the parental strain. This suggests that besides the altering of the electrostatic properties of the cell surface, the lack of D-alanylation may produce other effects that negatively impact Lys11 lytic action; these could be, for example, changes in cation availability in the CW and TA conformation [58,59,60].

The assays with *S. aureus* cells having altered CW glycopolymers therefore confirmed the WTA as a major determinant of Lys11 tolerance. Here, we uncovered more details of the WTA function as inhibitor of Lys11 binding to cells by showing that it hinders primarily the binding activity of CBD_11_. In fact, when cells have low levels of WTA, the Ami_11_ domain is almost dispensable both for binding and cell lysis. Yet, the available data indicate that the inhibitory action of WTAs goes beyond a simple shielding effect that restricts endolysin binding to the cell surface [24]. WTAs might also exclude endolysins from certain regions of the CW and contribute to the inhibition of catalytic activity due to their interplay with the PMF [42,57].

## 4. Materials and Methods

### 4.1. Bacterial Strains and General Growth Conditions

*Escherichia coli* and *S. aureus* strains used in this work are listed in Table 1. Unless stated otherwise, *E. coli* and *S. aureus* were grown at 37 °C, under aerated conditions, in Lysogeny Broth (LB, NZYTech—Genes & Enzymes) and tryptic soy broth (TSB, BIOKAR Diagnostics) media, respectively. When necessary for strain/plasmid selection, LB was supplemented with 100 μg/mL ampicillin and/or 40 μg/mL kanamycin and TSB with 120 µg/mL spectinomycin. Specific growth conditions for protein production in *E. coli* are described in Section 4.3. To generate a phenotype of impaired WTA synthesis without significantly affecting growth rate, *S. aureus* was cultured in presence of 50 ng/mL tunicamycin [24].

### 4.2. Generation of Endolysin Variants

Plasmids expressing Lys11 derivatives were constructed following standard recombinant DNA techniques. The endolysin variants produced and purified in this work are indicated in Table 2. Previously described derivatives of the expression vector pIVEX2.3d (Roche Applied Science, Mannhein, Germany) carrying Lys11 and eGFP coding sequences [21,24] were the basis for generating new endolysin variants (Supplementary File S1 and Figure S2). Genes expressing domain deletion mutants or domain fusions to eGFP were assembled by PCR or overlap-extension PCR, using plasmids carrying *lys11* and *eGFP* as templates, and suitable primers (Table S1). All coding sequences were inserted in pIVEX2.3d with *Nco*I and *Xma*I restriction sites, allowing variants to be tagged at the C-terminus with a hexahistidine tail. The recombinant plasmids, selected in presence of 100 μg/mL ampicillin, were confirmed by sequencing before transformation of *E. coli* expression strain CG61.

### 4.3. Protein Production and Purification

Two previously described protein production conditions [24] were applied according to the group of proteins, with group A including Lys11, CHAP_11_-CBD_11_ and Ami_11_-CBD_11_ and group B including eGFP-Ami_11_-CBD_11_, eGFP-Ami_11_ and eGFP-CBD_11_. Irrespective of the protein group, the different *E. coli* CG1 derivatives were grown overnight at 28 °C and on the next day 100-fold diluted either in phosphate-buffered LB medium supplemented with 0.5 M D-sorbitol (group A) or in regular LB (group B). Cultures were grown at 28 °C until mid/late exponential phase, and then protein production was induced by temperature upshift (30 min at 42 °C in a shaking water bath). Cultures from group A proteins were then incubated at 16 °C for 14–16 h, whereas those of group B were incubated for 3 h at 37 °C. After cell disruption [24], Lys11 deletion mutants and eGFP fusions were purified by metal chelate affinity chromatography, as reported previously for Lys11 and eGFP-Ami_11_-CBD_11_, respectively [24], except that all buffers contained 30% glycerol to minimize protein precipitation (Figure S3). Protein quantification and storage were also as described before [24].

### 4.4. Bacteriolysis Assays

*S. aureus* cell lysis as a result of lysin treatment in liquid culture medium was studied essentially as before [24]. Briefly, cells from exponentially growing cultures were collected by centrifugation and resuspended in fresh, pre-warmed TSB supplemented with 0.5 mM CaCl_2_ (TSBca) to an initial OD_600nm_ of ~0.8 (cuvette with slit and light path of 0.5 and 1 cm, respectively), which corresponded to about 1 × 10^8^ CFU/mL. Cells were challenged with Lys11 (or its derivatives) after 10 min treatment with the ionophores gramicidin (30 µg/mL, Sigma-Aldrich, Saint Louis, Missouri, USA, Cat. No. G5002), nigericin (10 µM, Sigma-Aldrich, Cat. No. N7143), valinomycin (20 µM, Sigma-Aldrich, Cat. No. V0627) or the corresponding ionophore solvents. These were DMSO (gramicidin and valinomycin) and ethanol (nigericin). Cells treated with valinomycin were also supplemented with 200 mM KCl. The ionophore treatments were defined based on previous work [30], with the drug concentrations adjusted to the minimum required to inhibit *S. aureus* growth in our experimental conditions. To monitor cell lysis, the OD_600nm_ of cultures in 96-well microplates (200 µL final volume per well) was measured at regular time points after addition of the different agents (Epoch 2 microplate reader, Biotek Instruments, Inc., Winooski, VT, USA). The impact of gramicidin, nigericin and valinomycin on the PMF was confirmed using the membrane potential-sensitive dye DiSC_3_(5) (Sigma-Aldrich, Cat. No. 43608), as reported previously [24].

*S. aureus* cell lysis in soft agar medium was studied using a spot assay. Cultures at an OD_600nm_ of ~0.8 were centrifuged, and cells were resuspended in 1/100 volumes of incorporation buffer (50 mM HEPES, 150 mM NaCl, 0.5 mM CaCl_2_, pH 7.2). A 300 µL sample of this suspension was added to 10 mL of soft agar incorporation buffer (0.75% agar) and poured into a Petri dish. Soft agar incorporation buffer was prepared by mixing equal volumes of 2× incorporation buffer and a solution of 1.5% agar (both solutions equilibrated at 50 °C before mixing). After solidification and drying, 10 µL drops of lysin dilutions in incorporation buffer were spotted onto the dense lawns of viable *S. aureus* cells. Plates were incubated overnight at 37 °C, and formation of lysis halos was analyzed.

### 4.5. Binding of eGFP-Endolysin Fusions to Cells

The binding of eGFP-endolysin fusions to *S. aureus* cells in different conditions was studied exactly as described in Gouveia et al. [24]. Cells of strain RN4220 set to an OD_600nm_ of ~0.8 in fresh TSBca were treated or not with ionophores (see Section 4.4), and then 200 µL cell samples were incubated with the indicated concentrations of fluorescent proteins for additional 10 min. After washing cells with PBS for removal of unbound protein, the fluorescence associated with cells was measured in black microtiter plates (Greiner Bio-One, Kremsmünster, Austria, Cat. No. 655076) with excitation and emission wavelengths of 488 and 507 nm, respectively (Varioskan LUX Multimode, ThermoFisher Scientific, Waltham, MA, USA). OD_600nm_ was also registered. The amount of eGFP-endolysin fusion associated with cells, expressed in nM.OD_600nm_^−1^, was calculated by performing standard calibration curves with each fluorescent protein as described previously [24]. The same method was employed to measure the binding of eGFP fusions to *S. aureus* RN4220 cells grown in the presence of 50 ng/mL tunicamycin.

### 4.6. Bioinformatics Analysis

Protein-conserved domains, domain boundaries and putative linker regions were defined using InterPro: https://www.ebi.ac.uk/interpro/ (accessed on 1 January 2023), CD-search: https://blast.ncbi.nlm.nih.gov/Blast.cgi (accessed on 1 January 2023) and the AlphaFold structure prediction for LytO (same as Lys11): https://alphafold.ebi.ac.uk/entry/Q2FX77 (accessed on 1 January 2023). Theoretical molecular masses, pI values and net charge at pH 7 (*z*) of recombinant proteins were determined with the Prot pi Protein tool: https://www.protpi.ch/Calculator/ProteinTool (accessed on 1 October 2023), using ExPASy as the data source of pKa values.

### 4.7. Statistical Analysis

Data are represented as the mean ± standard deviation from at least 3 independent experiments and were analyzed using GraphPad Prism version 10.0.2 (Boston, MA, USA). Data normality was confirmed with Kolmogorov–Smirnov test. For multiple group comparisons, the significance of the data differences was analyzed with a two-way ANOVA test, followed by Tukey’s post hoc test. Two group comparisons were made using Student’s *t*-test. Differences were considered statistically significant when the calculated adjusted *p* value was below the alpha level of 0.05.

## 5. Conclusions

In summary, the results of the present study reinforce the notion that the roles of the PMF and WTA in endolysin tolerance are most likely multifactorial and interconnected. By influencing the electrochemical environment of the CW, the PMF directly impacts endolysins by interfering with their binding and catalytic activities while probably also modulating the charge/conformation of WTAs. On the other hand, WTAs not only hinder endolysin binding but are also likely to contribute to the inhibitory role of the ΔpH component of the PMF by retaining protons in the inner layers of the CW. When thinking about the natural context of the action of endolysins, that is, during phage infection, it is relevant to note that the lytic enzymes only act after complete PMF dissipation mediated by the holin [10] and that this event can also stimulate endolysin lytic action from within [21]. This, associated with the fact that PMF collapse is also linked to the activation of bacterial autolysins [57,65], confirms the pivotal role of the PMF in the control of many peptidoglycan-degrading enzymes.

## Figures and Tables

**Table 1:** *Escherichia coli* and *S. aureus* strains used in this study.

Strains	Relevant Features	Reference/Source
*E. coli*		
XL1-Blue MRF’	Cloning strain for recovering of plasmid constructs	Stratagene
CG61	Protein expression strain; produces phage T7 RNA polymerase upon thermal induction. Used to produce all Lys11 variants. Selection with 40 μg/mL kanamycin	[61]
*S. aureus*		
RN4220	Prophage-cured, restriction-deficient mutant of strain 8325-4	[62]
RN4220Δ*tarM*Δ*tarS*	Derivative of RN4220 lacking α- and β-O-GlcNAcylation due to *tarM* and *tarS* deletion	[33]
RN4220Δ*spa*	In-frame deletion in *spa* coding for protein A	[63]
RN4220Δ*spa*Δ*ltaS* (supressor strain 4S5)	Derivative of RN4220Δ*spa* lacking LTA as result of *ltaS* deletion. Carries a mutation suppressing the Δ*ltaS* lethal phenotype	[32]
SA113	Mutant strain of 8325, with an agr^-^ background and 11-bp deletion in *rsbU*	[64]
SA113Δ*dltA*	Derivative of SA113 lacking D-alanylation of TA due to *dltA* deletion. Selection with 120 µg/mL spectinomycin	[34]

**Table 2:** Variants of endolysin Lys11 used in this study.

Lys11 Variant	Features	Reference/Source
Lys11	3-domain endolysin: CHAP_11_, Ami_11_ and CBD_11_	[21]
CHAP_11_-CBD_11_	Lys11 lacking Ami_11_. Deletion encompassing residues 151 to 360 of Lys11	This work
Ami_11_-CBD_11_	Lys11 lacking CHAP_11_. Deletion encompassing residues 2 to 178 of Lys11	This work
eGFP-Ami_11_-CBD_11_	Ami_11_-CBD_11_ (P_149_ to S_481_ of Lys11) fused to the C-terminus of eGFP	[24]
eGFP-Ami_11_	Ami_11_ (P_149_ to M_360_ of Lys11) fused to the C-terminus of eGFP	This work
eGFP-CBD_11_	CBD_11_ (D_361_ to S_481_ of Lys11) fused to the C-terminus of eGFP	This work

## Data Availability

The data presented in this study are openly available in FigShare at 10.6084/m9.figshare.24747519, reference number [66].

## Supplementary Materials

The following supporting information can be downloaded at https://www.mdpi.com/article/10.3390/ijms25010523/s1.
