# Supplementary material for: Cellular and Enzymatic Determinants Impacting the Exolytic Action of an Anti-Staphylococcal Enzybiotic
Source: Int J Mol Sci. 2023 Dec 30;25(1):523. doi: 10.3390/ijms25010523 (PMC10778630; doi:10.3390/ijms25010523)
Supplement: Supplementary file 1 [file ijms-25-00523-s001.zip › Supplementary Figures S1-S4_Revision2.pdf]

Supplementary Figures to:

Cellular and Enzymatic Determinants Impacting the Exolytic Action of an Anti-Staphylococcal Enzybiotic

Ana Gouveia [1] , Daniela Pinto [1,†,‡] , Jorge M. B. Vítor [2] and Carlos São-José [1,*]

[1] Phage Biology Research and Infection Control (PhaBRIC), Research Institute for Medicines (iMed.ULisboa), Faculdade de Farmácia, Universidade de Lisboa, Av. Prof. Gama Pinto, 1649-003 Lisboa, Portugal; ana.isabel.gouveia@ff.ulisboa.pt (A.G.); dspinto@ciencias.ulisboa.pt (D.P.)

[2] Pathogen Genome Bioinformatics and Computational Biology, Research Institute for Medicines (iMed.ULisboa), Faculdade de Farmácia, Universidade de Lisboa, Av. Prof. Gama Pinto, 1649-003 Lisboa, Portugal; jvitor@ff.ulisboa.pt

* Correspondence: csaojose@ff.ulisboa.pt

[†] Current address: Centre for Ecology, Evolution and Environmental Changes (cE3c) & CHANGE —Global Change and Sustainability Institute, Faculdade de Ciências, Universidade de Lisboa, 1749-016 Lisboa, Portugal.

[‡] Current address: Biosystems & Integrative Sciences Institute (BioISI), Faculdade de Ciências, Universidade de Lisboa, Campo Grande, 1749-016 Lisboa, Portugal.

Figure S1. Effect of ionophores on *S. aureus* membrane potential and cell viability

Figure S2. Schematic representation and features of the endolysin Lys11 variants used in this study

Figure S3. SDS-PAGE analysis of the purified Lys11 variants used in this work

Figure S4. The endolysin variant Ami_{11}-CBD_{11} displays no lytic action in liquid cultures

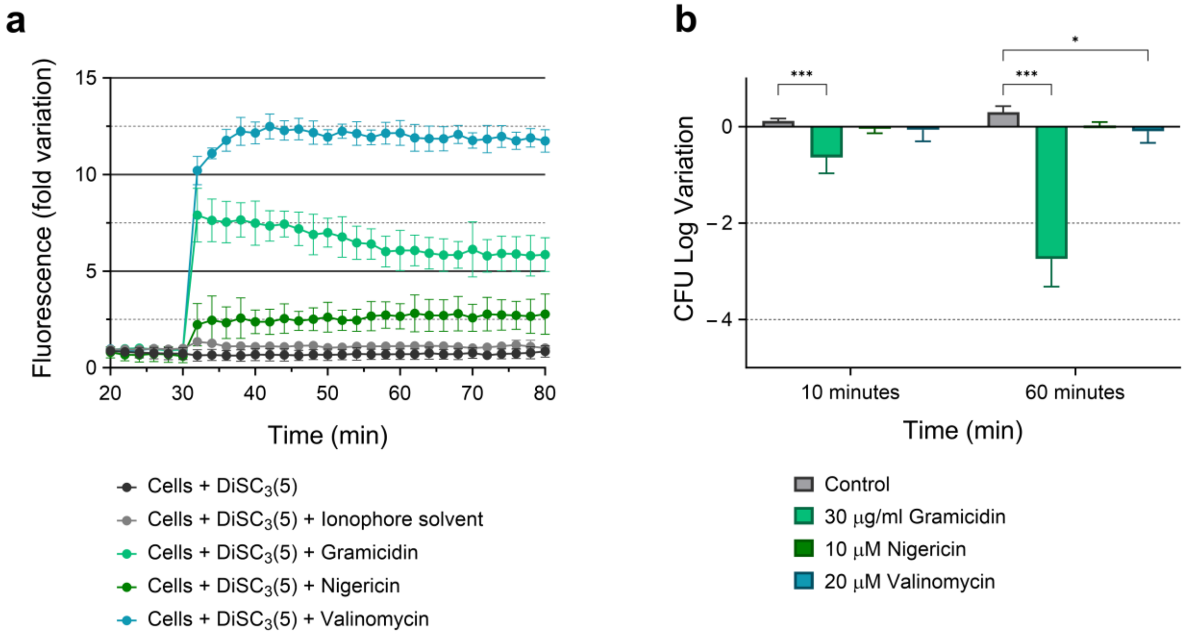

Figure S1. Effect of ionophores on *S. aureus* membrane potential and cell viability. **(a)** The expected effect of selected ionophores on the membrane potential of *S. aureus* cells was confirmed using the potentiometric fluorescence probe DiSC$_3$(5), as described previously [24]. DiSC$_3$(5) was added to polarized cells of strain RN4220, which uptake the dye and quenched its fluorescence. DiSC$_3$(5)-loaded cells were then treated with 30 µg/ml gramicidin, 10 µM nigericin, 20 µM valinomycin or ionophore solvent, and the increase in fluorescence as result of membrane depolarization monitored by fluorometry. At the tested concentrations, valinomycin and gramicidin caused an abrupt and marked rise in florescence, while nigericin produced only a slight increase, showing that the latter ionophore essentially worked as an electroneutral carrier. The data of each curve represent means ± standard deviation from at least 3 independent experiments. **(b)** Log phase cells of *S. aureus* strain RN4220 collected in fresh TSBca (see Section 4.4 in main text) were incubated at 37 °C for 10 or 60 min with the indicated concentrations of ionophores or solvent volume equivalents (Control). After incubation, cell viability was assessed by CFU counts. The results confirmed that significant lethality is only observed upon dissipation of both gradients of the PMF (gramicidin). For each condition, the results are represented as the log variation of CFU/ml relatively to the cell input. The data represents means ± standard deviation from at least 3 independent experiments. Asterisks denote a significant difference, according to two-way ANOVA, followed by Tukey post-hoc test (***p<0.001).

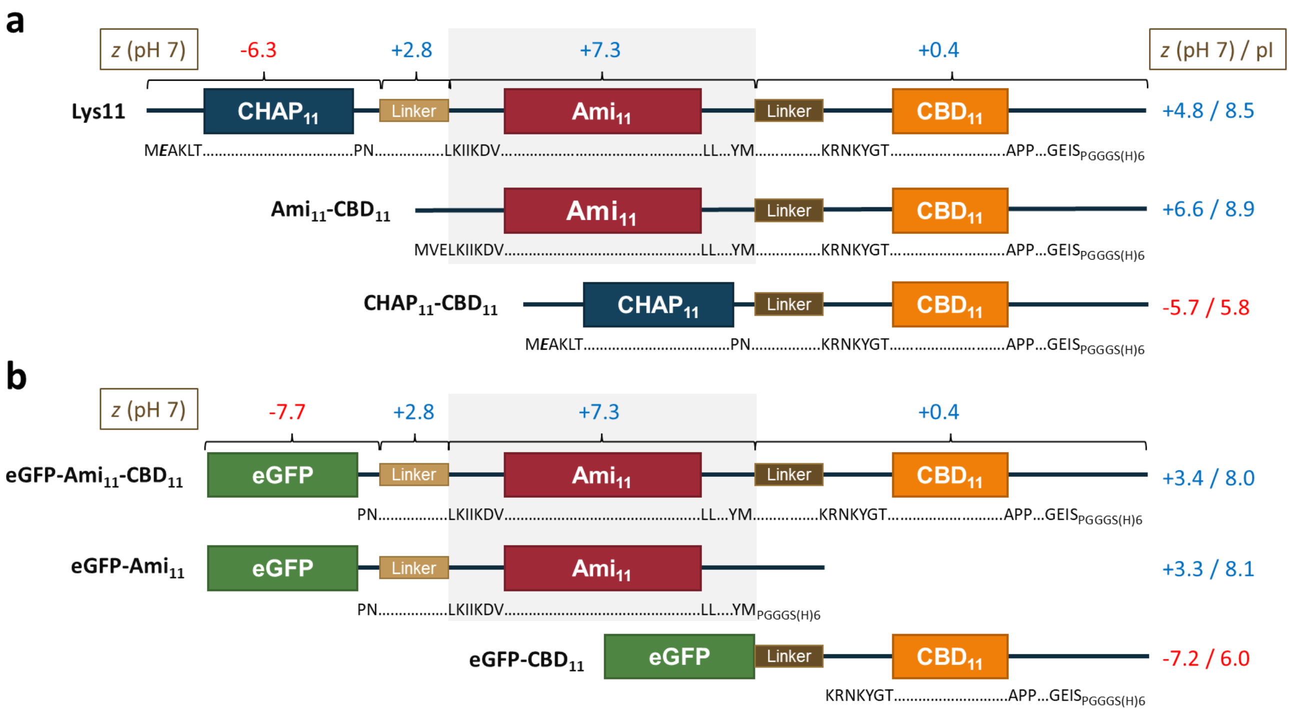

Figure S2. Schematic representation and features of the endolysin Lys11 variants used in this study (not drawn to scale). Relevant modules of deletion mutants **(a)** and eGFP fusions **(b)** are depicted with indication of the predicted net charge (*z* value) at pH 7. The *z* value and isoelectric point (pI) of the full-length constructs are also indicated on the right side. The *z* and pI values were predicted with the Prot pi Protein tool available at https://www.protpi.ch/Calculator/ProteinTool, using ExPASy as the data source for pKa values.

Gouveia *et al*.

Cellular and enzymatic determinants impacting the exolytic action of an anti-staphylococcal enzybiotic

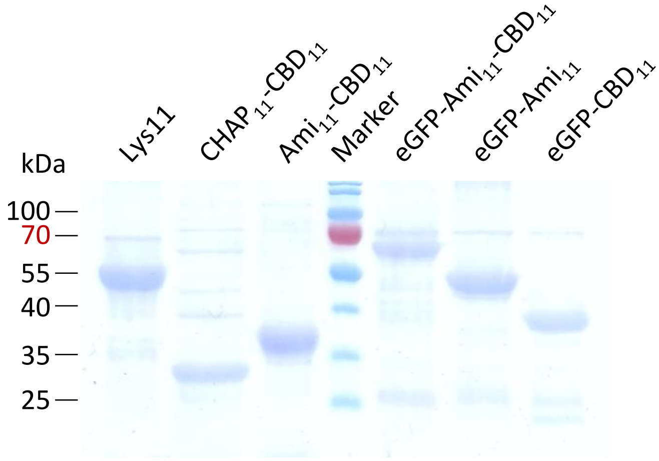

Figure S3. SDS-PAGE analysis of the purified Lys11 variants used in this work. The His_6-tagged recombinant proteins were purified by metal chelate affinity chromatography, followed by a desalting step to remove imidazole from the pure fractions. Each lane was loaded with 5 μg total protein. Molecular weight marker: PageRuler Prestained Protein Ladder (ThermoScientific).

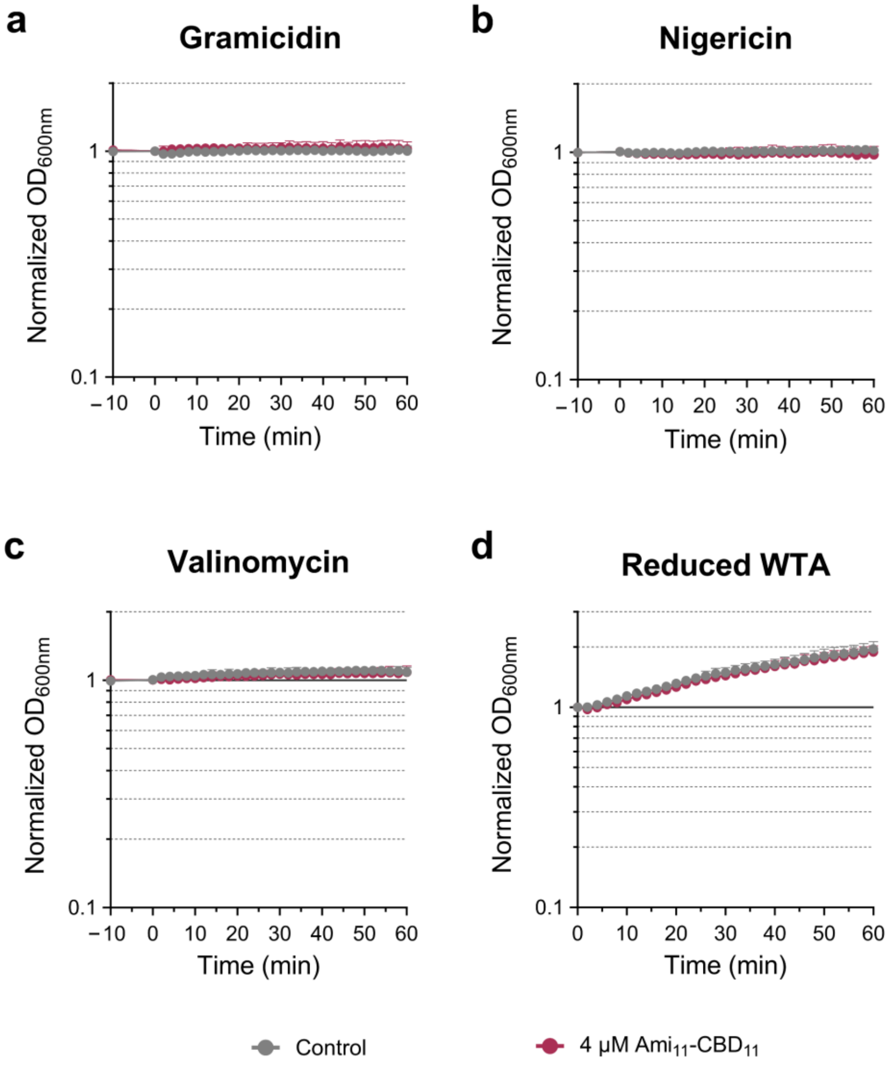

Figure S4. The endolysin variant Ami_{11}-CBD_{11} displays no lytic action in liquid cultures. *S. aureus* cells with affected PMF or with low WTA remain refractory to bacteriolysis mediated by Ami_{11}-CBD_{11}. Cells of strain RN4220 in TSBca were treated with gramicidin **(a)**, nigericin **(b)** or valinomycin **(c)**. Following ionophore treatment, 4 µM of Ami_{11}-CBD_{11} were added, and cell lysis monitored. **(d)** Cells of strain RN4220 grown in presence of tunicamycin were collected in TSBca, challenged with 4 µM of Ami_{11}-CBD_{11}, and lysis similarly monitored. In each panel, the "Control" curve corresponds to cells with ionophore or tunicamycin only (no protein added). Each curve represents means ± standard deviation from 4 independent experiments. For clarity, only the mean + standard deviation is represented
