# Supplementary material for: Cellular and Enzymatic Determinants Impacting the Exolytic Action of an Anti-Staphylococcal Enzybiotic
Source: Int J Mol Sci. 2023 Dec 30;25(1):523. doi: 10.3390/ijms25010523 (PMC10778630; doi:10.3390/ijms25010523)
Supplement: Supplementary file 1 [file ijms-25-00523-s001.zip › Supplementary File S1_Revision2.pdf]

Gouveia et al., 2023
Cellular and enzymatic determinants impacting the exolytic action of an anti-staphylococcal enzybiotic

Features of the endolysin Lys11 and derivatives used in this study

Relevant features of Lys11 and its derivatives are denoted in the corresponding primary sequences with colored lettering and shading.

Theoretical molecular masses, pI values and net charge at pH 7 (z) of Lys11 and its variants were determined with the Prot pi Protein tool (https://www.protpi.ch/Calculator/ProteinTool), using ExPASy as the data source of pKa values.

- **Protein domains**, according to InterPro (https://www.ebi.ac.uk/interpro/):

    CHAP (IPR007921), here designated $CHAP_{11}$

    Amidase (IPR002502), here designated Ami_{11}

    SH3-like (IPR003646), here designated CBD_{11}

    eGFP (IPR000786)

- **Linkers** were defined based on an integrated analysis with InterPro (for domain boundaries) and AlphaFold structure prediction of LytO (same as Lys11, https://alphafold.ebi.ac.uk/entry/Q2FX77).

- PGGGSHHHHHH is the vector-borne C-terminal tail containing the hexahistidine tag.

- "***E***" denotes a Q_2E substitution introduced in the native Lys11 sequence resulting from the use of the restriction enzyme *Nco*I recognition sequence in the cloning procedures.

Endolysin Lys11

>Lys11 (55.21 kDa, pI = 8.49, z =+4.79)
M***E***AKLTKNEFIEWLKTSEGKQFNVDLWYGFQCFDYANAGWKVLFGLLLKGLGAKDIPFANNFDGLATVYQNTP
DFLAQPGDMVVFGSNYGAGYGHVAWVIEATLDYIIVYEQNWLGGGWTDGIEQPGWGWEKVTRRQHAYDFPMWF
IRPNFKSETAPRSVQSPTQAPKKETAKPQPKAVELKIIKDVVKGYDLPKRGSNPKGIVIHNDAGSKGATAEAY
RNGLVNAPLSRLEAGIAHSYVSGNTVWQALDESQVGWHTANQIGNKYYYGIEVCQSMGADNATFLKNEQATFQ
ECARLLKKWGLPANRNTIRLHNEFTSTSCPHRSSVLHTGFDPVTRGLLPEDKRLQLKDYFIKQIRAYMDGKIP
VATVSNESSASSNTVKPVASAWKRNKYGTYYMEESARFTNGNQPITVRKVGPFLSCPVGYQFQPGGYCDYTEV
MLQDGHVWVGYTWEGQRYYLPIRTWNGSAPPNQILGDLWGEISPGGGSHHHHHH

Lys11 catalytic domain deletion mutants

>$CHAP_{11}$-CBD_{11} (31.89 kDa, pI = 5.83, z = -5.72)
M***E***AKLTKNEFIEWLKTSEGKQFNVDLWYGFQCFDYANAGWKVLFGLLLKGLGAKDIPFANNFDGLATVYQNTP
DFLAQPGDMVVFGSNYGAGYGHVAWVIEATLDYIIVYEQNWLGGGWTDGIEQPGWGWEKVTRRQHAYDFPMWF
IRPNDGKIPVATVSNESSASSNTVKPVASAWKRNKYGTYYMEESARFTNGNQPITVRKVGPFLSCPVGYQFQP
GGYCDYTEVMLQDGHVWVGYTWEGQRYYLPIRTWNGSAPPNQILGDLWGEISPGGGSHHHHHH

>Ami_{11}-CBD_{11} (35.15 kDa, pI = 8.93. z = +6.61)
MVELKIIKDVVKGYDLPKRGSNPKGIVIHNDAGSKGATAEAYRNGLVNAPLSRLEAGIAHSYVSGNTVWQALD
ESQVGWHTANQIGNKYYYGIEVCQSMGADNATFLKNEQATFQECARLLKKWGLPANRNTIRLHNEFTSTSCPH

RSSVLHTGFDPVTRGLLPEDKRLQLKDYFIKQIRAYMDGKIPVATVSNESSASSNTVKPVASAWKRNKYGTYY
MEESARFTNGNQPITVRKVGPFLSCPVGYQFQPGGYCDYTEVMLQDGHVWVGYTWEGQRYYLPIRTWNGSAPP
NQILGDLWGEISPGGGSHHHHHH

eGFP fusions to Lys11 domains

>eGFP-Ami$_{11}$-CBD$_{11}$ (65.17 kDa, pI = 7.99, z = +3.40)
MVSKGEELFTGVVPILVELDGDVNGHKFSVSGEGEGDATYGKLTLKFICTTGKLPVPWPTLVTTLTYGVQCFS
RYPDHMKQHDFFKSAMPEGYVQERTIFFKDDGNYKTRAEVKFEGDTLVNRIELKGIDFKEDGNILGHKLEYNY
NSHNVYIMADKQKNGIKVNFKIRHNIEDGSVQLADHYQQNTPIGDGPVLLPDNHYLSTQSALSKDPNEKRDHM
VLLEFVTAAGITLGMDELYKPNFKSETAPRSVQSPTQAPKKETAKPQPKAVELKIIKDVVKGYDLPKRGSNPK
GIVIHNDAGSKGATAEAYRNGLVNAPLSRLEAGIAHSYVSGNTVWQALDESQVGWHTANQIGNKYYYGIEVCQ
SMGADNATFLKNEQATFQECARLLKKWGLPANRNTIRLHNEFTSTSCPHRSSVLHTGFDPVTRGLLPEDKRLQ
LKDYFIKQIRAYMDGKIPVATVSNESSASSNTVKPVASAWKRNKYGTYYMEESARFTNGNQPITVRKVGPFLS
CPVGYQFQPGGYCDYTEVMLQDGHVWVGYTWEGQRYYLPIRTWNGSAPPNQILGDLWGEISPGGGSHHHHHH

>eGFP-Ami$_{11}$ (51.65 kDa, pI = 8.10, z = +3.33)
MVSKGEELFTGVVPILVELDGDVNGHKFSVSGEGEGDATYGKLTLKFICTTGKLPVPWPTLVTTLTYGVQCFS
RYPDHMKQHDFFKSAMPEGYVQERTIFFKDDGNYKTRAEVKFEGDTLVNRIELKGIDFKEDGNILGHKLEYNY
NSHNVYIMADKQKNGIKVNFKIRHNIEDGSVQLADHYQQNTPIGDGPVLLPDNHYLSTQSALSKDPNEKRDHM
VLLEFVTAAGITLGMDELYKPNFKSETAPRSVQSPTQAPKKETAKPQPKAVELKIIKDVVKGYDLPKRGSNPK
GIVIHNDAGSKGATAEAYRNGLVNAPLSRLEAGIAHSYVSGNTVWQALDESQVGWHTANQIGNKYYYGIEVCQ
SMGADNATFLKNEQATFQECARLLKKWGLPANRNTIRLHNEFTSTSCPHRSSVLHTGFDPVTRGLLPEDKRLQ
LKDYFIKQIRAYMPGGGSHHHHHH

>eGFP-CBD$_{11}$ (41.50 kDa, pI = 6.01, z = -7.19)
MVSKGEELFTGVVPILVELDGDVNGHKFSVSGEGEGDATYGKLTLKFICTTGKLPVPWPTLVTTLTYGVQCFS
RYPDHMKQHDFFKSAMPEGYVQERTIFFKDDGNYKTRAEVKFEGDTLVNRIELKGIDFKEDGNILGHKLEYNY
NSHNVYIMADKQKNGIKVNFKIRHNIEDGSVQLADHYQQNTPIGDGPVLLPDNHYLSTQSALSKDPNEKRDHM
VLLEFVTAAGITLGMDELYKDGKIPVATVSNESSASSNTVKPVASAWKRNKYGTYYMEESARFTNGNQPITVR
KVGPFLSCPVGYQFQPGGYCDYTEVMLQDGHVWVGYTWEGQRYYLPIRTWNGSAPPNQILGDLWGEISPGGGS
HHHHHH
